# The Development of a Multiplex PCR Assay for Fast and Cost-Effective Identification of the Five Most Significant Pathogenic *Prototheca* Species

**DOI:** 10.3390/pathogens12081018

**Published:** 2023-08-07

**Authors:** David Vasco-Julio, María Huilca-Ibarra, Yanua Ledesma, Gustavo Echeverria, Salome Guerrero-Freire, Tomasz Jagielski, Carlos Bastidas-Caldes, Jacobus H. de Waard

**Affiliations:** 1 One Health Research Group, Universidad de Las Américas, Quito 170530, Ecuador; david.vasco.julio@outlook.com (D.V.-J.); paula.huilca@outlook.com (M.H.-I.); yanua.ledesma@udla.edu.ec (Y.L.); carlos.bastidas@udla.edu.ec (C.B.-C.); 2 Posgrado en Ciencias Biológicas, Universidad Nacional Autónoma de México, Unidad de Posgrado, Edificio D, Circuito de Posgrados, Ciudad Universitaria, Coyoacán C.P. 04510, Mexico; 3 Centro de Investigación Sobre Enfermedades Infecciosas, Instituto Nacional de Salud Pública, Cuernavaca C.P. 62050, Mexico; 4 Instituto de Investigación en Zoonosis-CIZ, Universidad Central del Ecuador, Quito 170518, Ecuador; gustavo_echeverria@live.com; 5 División Investigación y Desarrollo, BioGENA, Quito 170509, Ecuador; 6 Programa de Doctorado, Facultad de Ciencias Veterinarias, Universidad de Buenos Aires, Buenos Aires C1063ACV, Argentina; salogue14@gmail.com; 7 Group of Emerging and Neglected Diseases, Ecoepidemiology and Biodiversity, Health Sciences Faculty, Universidad Internacional SEK, Quito 170521, Ecuador; 8 Department of Medical Microbiology, Institute of Microbiology, Faculty of Biology, University of Warsaw, 02-096 Warsaw, Poland; 9 INABIO—Instituto Nacional de Biodiversidad, Parque La Carolina, Quito 170135, Ecuador

**Keywords:** *Prototheca* genus, multiplex PCR (m-PCR), identification

## Abstract

A multiplex PCR system (m-PCR) has been developed to accurately differentiate the five most important pathogenic *Prototheca* species, including the three species associated with infection in dairy cattle (*P. ciferrii*, *P. blaschkeae*, and *P. bovis*) and the two species associated with human infections (*P. wickerhamii* and *P. cutis*). The method is low-cost since it employs a simple “heat-shock” method in a TE buffer for DNA extraction. Furthermore, it requires only primers, a Taq polymerase, an agarose gel, and a molecular weight marker for identification. The method was based on published *Prototheca* cytochrome B sequences and was evaluated using reference strains from each of the five *Prototheca* species. The validity of the method was confirmed by identifying 50 strains isolated from milk samples. The specificity was tested in silico and with experimental PCR trials, showing no cross-reactions with other *Prototheca* species, as well as with bacteria, fungi, cows, algae, animals, or humans. The method could detect mixed infections involving two or three *Prototheca* species, providing a rapid test that delivers results within three hours.

## 1. Introduction

*Prototheca* is a genus of free-living, achlorophilic microalgae that belongs to the *Chlorellaceae* family within the Chlorellales order [1]. These ubiquitous microorganisms can be found in soil and aqueous environments. In some cases, due to the loss of photosynthetic activity and their adaptation to heterotrophic growth, they can cause protothecosis infections in both animals and humans [2,3].

Bovine mastitis is a major form of animal protothecosis that can affect the entire herd, causing significant economic losses in the dairy industry [4,5,6]. The most common causative agents of bovine mastitis are *P. ciferrii*, *P. bovis*, and *P. blaschkeae* [4].

Pathogenicity in humans is only observed under certain conditions, such as a weakened or reduced host immune response level [2,7], and is mainly caused by *P. wickerhamii* and *P. cutis* [8]. However, species associated with infection in dairy cattle, such as *P. ciferrii* and *P. bovis*, have also been mentioned as causing human infections. It is important to note that the diagnosis of *Prototheca* infections can be challenging, as the symptoms can be similar to those of other diseases and the organism is often not suspected as a cause of infection. It may be confused with yeast or not detected in culture due to slow growth in the culture medium.

Several review articles provide an overview of *Prototheca* infections in both animals and humans, including the clinical manifestations, diagnosis, and treatment of the disease in different host species [3,9,10,11,12].

*Prototheca* infections can be diagnosed through culture from clinical samples, such as milk, urine, or tissue biopsy. Culture permits species-level identification and is particularly useful for epidemiological studies or identifying sources of infection. *Prototheca* species have simple nutritional requirements and grow readily on a variety of media within 72 h, although some slow-growing strains may require up to six days of incubation [8]. Species identification is typically based on macroscopic and microscopic examination, growth temperature, and patterns of sugar and alcohol assimilation [6,13,14].

In addition, various molecular methods have been developed to detect *Prototheca* DNA in clinical samples. These include endpoint PCR [7,15] or qPCR [16,17,18], but these methods often cannot identify the species. Other techniques, such as MALDI-MS [19], ITS amplification sequencing [20], cytochrome B (*CYTB*) sequencing [21], or PCR-RFLP analysis of the *CYTB* gene [22], can provide species-level identification. However, these techniques may pose challenges in terms of cost, time requirements, and usability, particularly in developing countries.

To address these challenges, multiplex PCR (m-PCR) could be an efficient approach for identifying the most important *Prototheca* species, particularly those that are significant in veterinary and human medicine, such as *P. ciferrii*, *P. bovis*, *P. blaschkeae*, *P. cutis*, and *P. wickerhamii*. By using an m-PCR that targets these key pathogenic species, we can potentially create a cost-effective and user-friendly tool for rapidly identifying the most significant *Prototheca* infections. In this study, we have designed specific primers for the detection of these five species. The identification process is achieved through a straightforward endpoint PCR procedure, followed by separation of the amplified fragments using an agarose gel.

## 2. Materials and Methods

### 2.1. Prototheca Species Used in the Study

All *Prototheca* strains utilized in this study were obtained from the culture collection of the Department of Medical Microbiology, Faculty of Biology, the University of Warsaw. The selected strains included the pathogenic species *P. ciferrii* and *P. bovis* (former *P. zopfii* genotypes 1 and 2), *P. blaschkeae*, *P. cutis*, *and P. wickerhamii*, alongside two environmental species, namely *P. stagnora* and *P. cookei.* In addition, a total of 50 *Prototheca* strains, isolated from bovine mastitis milk, were used for validation purposes.

### 2.2. Culture of Prototheca Species

To prevent bacterial contamination, *Prototheca* spp. were cultured on Sabouraud Dextrose Agar (SDA) supplemented with vancomycin (5 mg/mL) and nalidixic acid (25 mg/mL), following the method described by Huilca-Ibarra et al., (2022) [5]. The strains were incubated at 37 °C for a period of three to seven days until visible growth was observed.

### 2.3. Molecular Techniques

#### 2.3.1. DNA Isolation of *Prototheca* Using the Heath-Shock Method

DNA was efficiently extracted from the colonies grown on SDA using a simple, cost-effective, and rapid heat-shock method. To initiate the process, three 1-mm loops of cells were resuspended in a 1.5-mL Eppendorf tube containing 200 µL of TE buffer (10 mM Tris-HCl and 1 mM EDTA, pH 8.0) and frozen at −20 °C for further processing. Upon thawing, the cell suspension was subjected to a heat treatment and heated to 95 °C for 5 min. This temperature ensured the denaturation of cellular components, facilitating the release of DNA molecules. Subsequently, a quick centrifugation step at 10,000× *g* for 1 min was used to separate the cellular debris and other particulate matter, enabling easy access to the DNA-containing supernatant. To ensure the preservation and stability of the extracted DNA, the supernatant was collected and stored at −20 °C. This storage condition guarantees the long-term integrity and usability of the DNA for subsequent analyses and experiments.

#### 2.3.2. Identification of the Reference Strains by *CYTB* Sequencing

To confirm the identity of the strains used for validation of the m-PCR method, a fragment of the cytochrome B gene was sequenced using species-specific primers: cytB_F1 (5′-GyGTwGAACAyATTATGAGAG-3′) and cytB_R2 (5′-wACCCATAArAArTACCATTCWGG-3′), as described in the protocol outlined by Jagielski et al., (2018) [21]. The PCR reaction mixture had a final volume of 15 μL, consisting of 7.5 μL (1×) of Promega GoTaq^®^ DNA Polymerase (Promega, Madison, WI, USA), 0.5 μL (0.2 μM) of each primer, and 1.0 μL of DNA template. The PCR program included an initial denaturation step at 95 °C for 3 min, followed by 35 amplification cycles consisting of denaturation at 95 °C for 30 s, annealing at 48 °C for 30 s, and extension at 72 °C for 30 s. A final extension step at 72 °C for 5 min was performed. To visualize the amplicons, electrophoresis was conducted on a 1% agarose gel containing SYBR Safe reagent, and the bands were visualized using a ChemiDoc™ Imaging System (Bio-Rad, Hercules, CA, USA). Subsequently, all amplicons were sequenced by Sanger sequencing using an ABI 3500xL Genetic Analyzer (Thermo Fisher Scientific, Waltham, MA, USA) with BigDye 3.1^®^ capillary electrophoresis matrix. To confirm the *Prototheca* species, we performed Blastn analysis using the obtained sequences.

### 2.4. Primer Design for the Multiplex PCR

Specific primers for the multiplex polymerase chain reaction (m-PCR) were designed to target the cytochrome B (*CYTB*) gene as the desired genetic marker. To accomplish this, the nucleotide sequences of all known *Prototheca* species were extracted from the NCBI nucleotide database (available at https://www.ncbi.nlm.nih.gov/nucleotide/ (accessed on 2 February 2022). The sequences obtained for all five species were aligned to establish zones with more differences or similarities using the Ugene software (available at https://ugene.net/ (accessed on 15 February 2022). Primer design was carried out using PrimerBlas (available at https://www.ncbi.nlm.nih.gov/tools/primer-blast/ (accessed on 2 March 2022). Various criteria were considered for primer selection:

- Ensuring no sequence identity with other species of the *Prototheca* genus or loci.
- Optimal primer length ranges from 18 to 25 base pairs.
- Melting temperature (Tm) between 50 and 60 °C.
- Guanine and cytosine (GC) content percentages fall between 40 and 60%.
- Compatibility with multiplex reactions in terms of melting temperature (a temperature difference of less than 5 °C).
- A minimum difference of 60 base pairs in fragment sizes between primers.
- Absence of secondary structures (hairpins, homodimers, or heterodimers) between primer pairs.

Characteristics of each primer, including potential tertiary structures, melt temperature, length, guanine, and cytokine content, were analyzed using OligoAnalyzer, an online tool available from Integrated DNA Technologies (accessible at https://www.idtdna.com/pages/tools/oligoanalyzer (accessed on 15 March 2022). To determine the specificity in silico, Blastn (available at https://blast.ncbi.nlm.nih.gov/Blast.cgi (accessed on 15 March 2022) was used for the initial verification of the identity, considering that each primer must bind only to the complementary region of the *CYTB* gene of a specific species and not in other possible genomic regions (mitochondria, plastid chromosomes) of the same species or other species, also taking into account that the primers were not complementary to genomes of organisms disease-related pathogens and similar symptoms caused by any of the five species of the genus *Prototheca* included in this study. In silico PCR was performed using the SnapGene software (version 6.0.2), provided by Insightful Science (available at snapgene.com (accessed on 29 March 2022), which also facilitated compatibility and identity validation with different *Prototheca* species and their organelles. Table 1 provides detailed information on the primer sequences for each species, the approximate fragment size, and the accession numbers of the *CYTB* sequences used for primer design.

### 2.5. Multiplex PCR

To perform the multiplex PCR, a final volume of 25 µL was used, consisting of 15.0 µL (1.2×) of Promega GoTaq^®^ DNA polymerase (Promega, Madison, WI, USA), 0.4 µL (0.16 µM) of each species-specific primer, and 1.0 µL of unquantified DNA that was extracted using the method described in the previous section. The amplification process involved an initial denaturation step at 95 °C for 3 min, followed by 30 cycles of denaturation at 94 °C for 30 s, annealing at 56 °C for 30 s, and extension at 72 °C for 30 s. The process ended with a final extension step at 72 °C for 5 min.

After amplification, the resulting amplicons were visualized on a 2% agarose gel (*w*/*v*) in 1× Tris-borate- EDTA (TBE) buffer and DNA dye SYBR Safe reagent (Invitrogen, Waltham, MA, USA). Electrophoresis was carried out at 90 V for 45 min using a Labnet Enduro Gel XL Electrophoresis System (Labnet International, Inc., Edison, NJ, USA), and the bands were visualized using a ChemiDoc™ Imaging System (BioRad, Hercules, CA, USA). The size of the bands was approximated using a TrackIt™50 bp DNA ladder (Invitrogen, Waltham, MA, USA).

## 3. Results

### 3.1. Molecular Confirmation of the Identity of the Reference Strains

A fragment of the cytochrome B sequence from the control strains of *Prototheca* was amplified and subsequently sequenced. The Nucleotide BLAST analysis yielded the following NCBI accession numbers for the strains: *P. blaschkeae*: MF163449; *P. wickerhamii*: MN794237.1; *P. ciferrii*: MF163464.1; *P. bovis*: MH389235.1; *P. cutis:* MF163453.1; *P. stagnora*: MF163455.1; and *P. cookei*: MK452792.1.

### 3.2. Gradient Optimization for PCR Assays of the m-PCR Primers

The annealing temperature for the five primer sets was optimized by testing a range of temperatures above and below the calculated Tm of the primers. Initially, individual primer sets were tested, and subsequently, a consensus temperature for all five primer sets was determined by testing a range of temperatures in a reaction that included all five species. The results were evaluated for the presence of nonspecific bands using agarose gel electrophoresis. After careful analysis, an optimum annealing temperature of 56 °C was chosen.

### 3.3. Specificity of the Primers in the m-PCR Assay

In silico analysis confirmed that the primer pairs did not produce any nonspecific PCR amplification products with other organisms. Similarly, in experimental testing, the primers did not amplify DNA from bacteria such as *Staphylococcus aureus* and *Escherichia coli* (species commonly isolated from milk samples of mastitis cows), human and cow DNA, fungi of the *Candida* genus (often isolated from milk samples of mastitis cows), other algae belonging to the genus *Chlorella*, or the *Prototheca* species *P. stagnora* and *P. cookei*.

### 3.4. Amplification of the Target Genes

**Figure 1:**
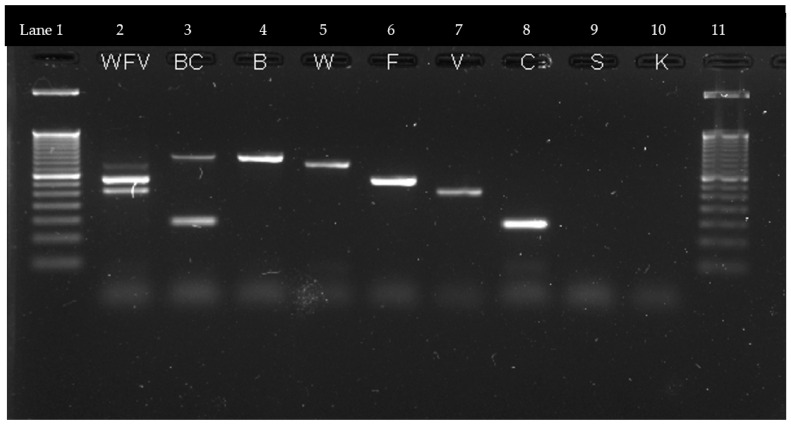
The m-PCR amplification profiles obtained in this study are displayed on a 2% agarose gel. The 50-bp molecular size ladder marker is present in lanes 1 and 11. The amplification of three species in the same reaction, namely *P. wickerhamii*, *P. ciferrii*, and *P. bovis*, is represented by the WFV bands. The BC bands correspond to the amplification of two species in the same reaction, specifically *P. blaschkeae* and *P. cutis*. The remaining lanes (4–8) show the individual amplification of each species, where all five primer pairs were used in each reaction. The resulting bands represent *P. blaschkeae* (B) (ca. 500 bp), *P. wickerhamii* (W) (ca. 425 bp), *P. ciferrii* (F) (ca. 350 bp), *P. bovis* (V) (ca. 250 bp), and *P. cutis* (C) (ca. 150 bp). In this experiment, the specificity was tested in lanes 9 and 10 with *P. stagnora* (S) and *P. cookei* (K) DNA, respectively. Both species showed no amplification product in the PCR reaction with the five primer sets, confirming the specificity of the assay.

The designed primer pairs, either individually or when used together in the same reaction, successfully amplified specific fragments of the *Prototheca* cytochrome B gene. Specifically, the amplification resulted in the generation of a 491 bp fragment for *P. blaschkeae*, a 425 bp fragment for *P. wickerhamii*, a 331 bp fragment for *P. ciferrii*, a 331 bp fragment for *P. bovis*, and a 267 bp fragment for *P. cutis* (Figure 1, lanes 4–8). The identity of these amplified fragments was confirmed by sequencing.

### 3.5. PCR Assay for Mixed Species Identification

When attempting to amplify the DNA of all five species in the same reaction, it was not possible to obtain amplification of all species simultaneously. However, when the DNA of three species was mixed together, three specific bands were observed, each corresponding to the respective species added to the reaction mix. See Figure 1, lanes 2 and 3. In lane 2 (WFV), the DNA of *P. wickerhamii*, *P. ciferrii*, and *P. bovis* was mixed, resulting in the appearance of three distinct bands on the gel. In lane 3 (BC), a coinfection simulation of *P. blaschkeae* and *P. cutis* was performed, showing the presence of two bands representing the two species.

### 3.6. Assay Validation

Fifty *Prototheca* isolates, obtained from milk samples, were subjected to identification using the m-PCR assay and cytochrome B sequencing. Both methods consistently identified all 50 isolates as *P. bovis*.

### 3.7. Sensitivity of the m-PCR Assay

The sensitivity of the m-PCR assay was not specifically determined, as its primary purpose is species identification rather than direct detection of pathogenic *Prototheca* species in clinical samples. However, sensitivity was investigated in the context of mixed infections. To evaluate this, we deliberately “contaminated” the DNA of one specific *Prototheca* species with DNA from another species. In these experiments, we were able to detect approximately 5% of the contaminating DNA in a PCR reaction.

## 4. Discussion

We have successfully developed a highly specific multiplex PCR assay capable of identifying the five most significant *Prototheca* species in a single reaction. This method requires the isolation and culture of the target species for identification. The DNA extraction protocol we employed is simple and efficient, involving a heat step and the use of TE buffer as the only required reagent. Within approximately 15 min, DNA extraction can be completed without the need for specialized lysis buffers, proteinase K, or DNA purification kits, which are commonly used in other studies on DNA extraction from *Prototheca* species [21].

### 4.1. DNA Isolation

The purified DNA, dissolved in TE buffer, was not quantified in our protocol. Instead, we used a consistent amount of DNA obtained from three one-mm loops suspended in 200 μL of TE buffer. This DNA solution consistently yielded satisfactory PCR products, with only 1 μL of this solution required as a template for PCR amplification. The m-PCR method is cost-effective since it only requires a DNA polymerase for amplification, and the detection step is carried out using a 15 mL 2% agarose gel. The entire process, from sample harvesting to obtaining results, can be completed within approximately three hours. This rapid turnaround time makes the m-PCR assay a valuable tool for the swift and early identification of *Prototheca* species.

### 4.2. Primers

The primers utilized in our assay target the *CYTB* gene, which has been demonstrated to be superior in differentiating and identifying *Prototheca* species when compared to other genes [21,23]. Through in silico tests and experimental trials, we have established the specificity of the primers, with no observed cross-reactions with other *Prototheca* species, bacteria, fungi, algae, plants, animals, or humans. This specificity is of particular significance in clinical settings where *Prototheca* cultures can be mistakenly identified as fungi due to their growth characteristics. The multiplex PCR method can rapidly identify the *Prototheca* species implicated in clinical cases, including those associated with cutaneous and systemic infections in both humans and animals.

### 4.3. Use of the m-PCR with Isolates from Milk Samples

We applied our developed multiplex PCR assay to analyze clinical samples of bovine mastitis that had previously been identified in Ecuador by Huilca-Ibarra et al., (2022) [5]. The results of our PCR analysis were consistent with the findings of the mentioned study. Sequencing of the amplified fragments confirmed that the causative species of mastitis in those samples was *P. bovis*, which was accurately identified using our multiplex PCR assay. This validation demonstrates the reliability and effectiveness of our assay for the identification of *Prototheca* species in bovine mastitis cases.

### 4.4. Detection of Mixed Infections

Concerning mixed infections, while it is true that all five *Prototheca* species cannot be identified in a single reaction, we contend that this limitation is inconsequential in clinical scenarios where the presence of multiple species is unlikely. The focus of our assay is to accurately identify the specific *Prototheca* species relevant to a given case, and in that regard, it has proven to be highly reliable and effective.

Another m-PCR assay has been previously reported, aiming at differentiation between three species: *P. bovis*, *P. blaschkeae*, and *P. wickerhamii*, which are known to cause bovine intramammary infection [24]. In our study, we expanded upon this assay by incorporating two additional species, *P. ciferrii* and *P. cutis*. This expansion allows for the assay’s applicability to human isolates, as *P. wickerhamii*, *P. cutis*, and *P. bovis* have also been documented to infect humans [8,9,25,26]. By including these additional species, our m-PCR assay offers a comprehensive approach for the identification of *Prototheca* species in both veterinary and human settings.

## 5. Limitations of this Study

To ensure the robustness and reliability of the m-PCR method, it is important to conduct further validation by examining additional strains that represent the five pathogenic *Prototheca* species. It should be noted that the present study only tested one strain per species that was readily available. Therefore, expanding the validation to include a broader range of strains will enhance the generalizability and applicability of the m-PCR assay. By including multiple strains for each species, we can better assess the assay’s performance across different genetic variations and ensure its accuracy and effectiveness in identifying pathogenic *Prototheca* species. During the development of this m-PCR assay, we did not perform a sensitivity analysis as our main objective was to achieve rapid identification of *Prototheca* species from isolated colonies. It is important to note that this m-PCR assay is not designed for the detection of *Prototheca* in clinical samples.

## Figures and Tables

**Table 1:** *Prototheca* species, primer sequences, fragment PCR product sizes, and NCBI access numbers used to develop the primers.

	Primer Pairs	Size (pb)	NCBI Access Number Cyt B
*P. blaschkeae*	F-bla: 5′-GCGGTTGGTTTTTACGTTAT-3′	491	MF163449
	R-bla: 5′-TGCAAAAACAGTCCATCCG-3′		
*P. wickerhamii*	F-wic: 5′-CATGTTCCGTGGTCTGTACT-3′	425	MF163461.1
	R-wic: 5′-TAGCGAAAGCTACTGTCCC-3′		
*P. ciferrii*	F-cif: 5′-AGCTAGTGCTATTCCAGTTG-3′	331	NC_037449.1
	R-cif: 5′-TGCAGGAATATAGTTATCAGGG-3′		
*P. bovis*	F-bov: 5′-CGCAACATTAAACCGTTTCT-3′	267	MH389235.1; MF163473.1
	R-bov: 5′-GGGTTCGCAGGAATGTAATTAT-3′		
*P. cutis*	F-cut: 5′-GGAAACCCACTAGGTGTAAG-3′	144	MF163453.1
	R-cut: 5′-CAGGATGTCCTAAAGCATTC-3′		

## Data Availability

All data generated or analyzed during the current study are included in this published article.
